# Ultrasonographic Characterization of the *db/db* Mouse: An Animal Model of Metabolic Abnormalities

**DOI:** 10.1155/2018/4561309

**Published:** 2018-03-08

**Authors:** Francesco Faita, Nicole Di Lascio, Chiara Rossi, Claudia Kusmic, Anna Solini

**Affiliations:** ^1^Institute of Clinical Physiology, Italian National Research Council, Pisa, Italy; ^2^Institute of Life Sciences, Scuola Superiore Sant'Anna, Pisa, Italy; ^3^Department of Clinical and Experimental Medicine, University of Pisa, Pisa, Italy; ^4^Department of Surgical, Medical, Molecular, and Critical Area Pathology, University of Pisa, Pisa, Italy

## Abstract

The availability of an animal model able to reliably mirror organ damage occurring in metabolic diseases is an urgent need. These models, mostly rodents, have not been fully characterized in terms of cardiovascular, renal, and hepatic ultrasound parameters, and only sparse values can be found in literature. Aim of this paper is to provide a detailed, noninvasive description of the heart, vessels, liver, and kidneys of the *db/db* mouse by ultrasound imaging. Sixteen wild type and thirty-four *db/db* male mice (11-week-old) were studied. State-of-the-art ultrasound technology was used to acquire images of cardiovascular, renal, and hepatic districts. A set of parameters describing function of the selected organs was evaluated. *db/db* mice are characterized by systolic and diastolic dysfunction, confirmed by strain analysis. Abdominal aortic and carotid stiffness do not seem to be increased in diabetic rodents; furthermore, they are characterized by a smaller mean diameter for both vessels. Renal microcirculation is significantly compromised, while liver steatosis is only slightly higher in *db/db* mice than in controls. We offer here for the first time an *in vivo* detailed ultrasonographic characterization of the *db/db* mouse, providing a useful tool for a thoughtful choice of the right rodent model for any experimental design.

## 1. Introduction

Clinicians are aware that a series of metabolic disorders (atherogenic dyslipidemia, hyperglycemia and insulin resistance, overweight or obesity, prothrombotic state, and arterial hypertension) tend to cluster in a single individual, thus conferring a high cardiovascular risk profile [1, 2]. C57BL/KSJ-*db/db* mice, carrying a mutation on the chromosome 4 that inhibits the expression of the leptin receptor (long isoform) and drives an impaired signal transduction [3], represent a validated animal model for the study of common metabolic abnormalities like obesity, dyslipidemia, and type 2 diabetes. Although results of these studies can be only partially translated to humans, due to profound differences in the pathophysiology of type 2 diabetes (a multifactorial disease with a complex interaction between genetic and environmental factors) respect to this genetic model [4], these mice are characterized by overweight, insulin resistance, and high plasma cholesterol and triglycerides and develop sustained hyperglycemia as early as six weeks after birth [5], whose occurrence is driven by a peripheral-impaired insulin action and a progressive *β*-cell failure [6]. They also display a hepatic steatosis that may progress to nonalcoholic steatohepatitis (NASH) [7], therefore representing an interesting animal model for studying the hepatic impairment following the above-reported metabolic abnormalities.

A relevant role in the complex pathophysiology of cardiovascular damage during the course of obesity and type 2 diabetes is certainly played by low-grade inflammation, strong promoter of atherosclerosis but also able to drive abnormalities in cardiac function and structure [8, 9]. Therefore, in performing animal studies, a deep knowledge of the animal phenotype becomes an urgent need, to better dissect the role of environmental determinants of vascular damage (e.g., high-fat diets, frequently used to accelerate the atherosclerotic process in these animals) or to explore the efficacy of novel treatments. This information should be ideally acquired through the application of noninvasive and safe methods that would allow the design of longitudinal multitime points studies and would improve the knowledge being, at the same time, fully respectful of the animal welfare.

In this view, high-frequency ultrasound (hfUS) represents the optimal imaging technique: it is not invasive, it does not require the sacrifice of the animal, it does not imply the use of ionizing agent's administration, and it provides multiorgan information without inducing any major discomfort in animals. However, the characterization of the *db/db* murine model obtained by ultrasound imaging is incomplete and, often, controversial.

In face of several reports dealing with the ultrasonographic description of the cardiovascular system, to the best of our knowledge, no data are available on *db/db* mice carotid stiffness evaluated *in vivo*, and only Reil et al. [10] have published aortic stiffness measurements obtained *in vivo* by magnetic resonance imaging, a complex, expensive, and scarcely available technique. Other papers report aortic stiffness of *db/db* mice [11, 12], but all these studies are based on ex vivo analysis of explanted vessels. On the other hand, information on renal and hepatic status in this animal model are scanty: renal dysfunction has been assessed by ultrasound in a murine model of myocardial infarction [13] and obesity [14], but no *in vivo* data are available on the *db/db* mice; furthermore, an assessment of the degree of steatosis evaluated *in vivo* by ultrasound in the *db/db* model has never been performed to the best of our knowledge.

The apparent contradiction or incompleteness of the literature could be ascribed to the adoption of different imaging technologies. In fact, some authors reported the use of standard clinical ultrasound devices, while others adopted more performant experimental high-resolution ultrasound systems.

With these considerations in mind, we designed a study aimed at offering, through a novel and technically advanced approach, a detailed noninvasive characterization of the liver, renal, and cardiovascular phenotype of the *db/db* mouse.

## 2. Materials and Methods

### 2.1. Experimental Protocol

The study was performed in accordance with the European Directive (2010/63/UE) and the Italian law (D.Lvo 26/2014), and it followed the principles of laboratory animal care. The Local Ethical Panel approved the protocol (n° 943/2015-PR).

Sixteen C57BL6 (wt) and thirty-four *db/db* 11-week-old male mice were purchased from Envigo RMS Srl, Udine, Italy. Animals were housed in standard cages and in temperature- and humidity-controlled quarters, with constant 12 : 12-h light-dark cycles. Mice were given Teklad Global 16% Protein Rodent Diet chow (Envigo RMS Srl, Udine, Italy) and water ad libitum and were acclimatized for two weeks before the study.

All the animals were weighted and underwent a hfUS examination with a high-resolution imaging system (Vevo 2100, FUJIFILM VisualSonics Inc., Toronto, Canada). Mice were anesthetized with isoflurane using an induction chamber connected with a scavenger canister. After induction, each mouse was placed on a temperature-controlled board, and the four limbs coated with conductive paste and taped on the ECG electrodes. During the examination, the animals were maintained under gaseous anesthesia by a nose cone (1.5% isoflurane in 1.0 l/min of pure oxygen), monitoring heart rate, respiration frequency, and body temperature. The abdomen was shaved and coated with acoustic coupling gel. A 24 MHz and 55 MHz hfUS probe (MS250 and MS550, FUJIFILM VisualSonics Inc., Toronto, Canada) held in position by a mechanical arm was used for the acquisitions. During all the examinations, ECG and respiration signals were acquired using the Advancing Physiological Monitoring Unit provided with the imaging station (Vevo Imaging Station, FUJIFILM VisualSonics Inc., Toronto, Canada).

### 2.2. Plasma Biochemical Analysis

Whole blood samples (250 *μ*l) were collected in K_2_-EDTA tubes from the tail vein the day after the ultrasonographic evaluation, to avoid pretest stress. Samples were centrifuged at 1000 ×g for 20 min at 4°C; and the plasma was used for determinations of glucose (glucose oxidase method), cholesterol and triglycerides, and aspartate aminotransferase (AST).

### 2.3. Cardiac Imaging and Assessment

**Figure 1:**
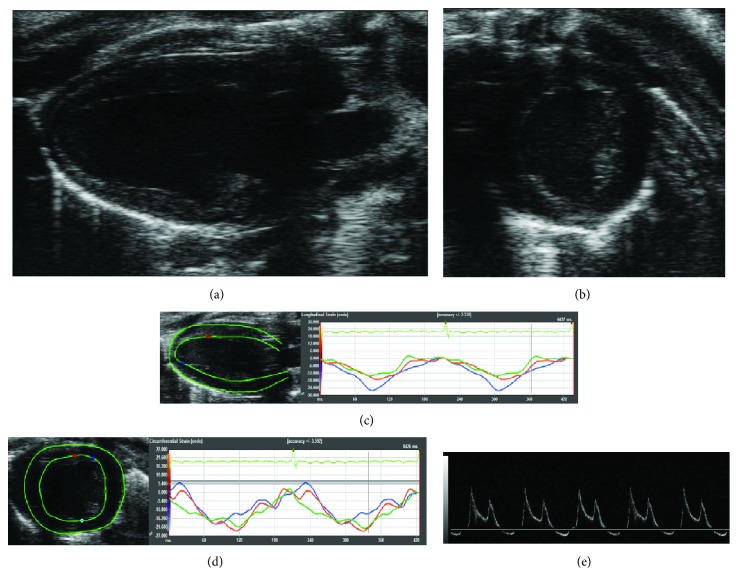
Cardiac ultrasound scan. (a) Parasternal long axis view of the left ventricle. (b) Parasternal short axis view of the left ventricle. (c) Analysis of the longitudinal and radial strain/strain rate. (d) Analysis of the circumferential and radial strain/strain rate. (e) Parasternal 4-chamber view with PW-Doppler for the evaluation of E/A parameter.

B-mode images of the heart were acquired with the higher frequency probe in parasternal long axis (PLAX) and short axis (SAX) views (Figures 1(a) and 1(b), resp.) and then analyzed offline to calculate left ventricular mass (LVmass), cardiac output (CO), fractional shortening (FS), stroke volume (SV), and ejection fraction (EF) from semiautomatic tracings of the ventricle borders in PLAX by means of the LV Trace software (FUJIFILM VisualSonics Inc., Toronto, Canada) [15]. Transmitral inflow pulsed wave Doppler (PW-Doppler) obtained in apical 4-chamber view (Figure 1(e)) was used for the evaluation of the LV diastolic function: ratio of the early to late ventricular filling velocities (E/A) was calculated by elaborating mitral inflow data and used as diastolic function parameter [16]. Strain analysis was performed on B-mode images acquired in both PLAX and SAX projections (Figures 1(c) and 1(d), resp.) using VevoStrain (FUJIFILM Visualsonics Inc., Toronto, Canada); this software is based on a speckle-tracking algorithm [17]. Briefly, for each recorded B-mode cine loop, two consecutive cardiac cycles were selected for analysis based on adequate visualization of the endocardial border and absence of image artifacts. Starting endocardial and epicardial contours were semiautomatically traced: the left ventricular myocardium was then automatically divided in six standard anatomical segments and the speckle-tracking algorithm was applied regionally, providing regional strain and strain rate curves. These curves were obtained for the longitudinal direction using PLAX B-mode images and for the radial and circumferential direction using SAX B-mode images. Longitudinal, radial, and circumferential global strains (gLS, gRS, and gCS) and strain rates (gLSR, gRSR, and gCSR) were calculated averaging the peak values of the corresponding curves across all six segments.

### 2.4. Vascular Imaging and Assessment

**Figure 2:**
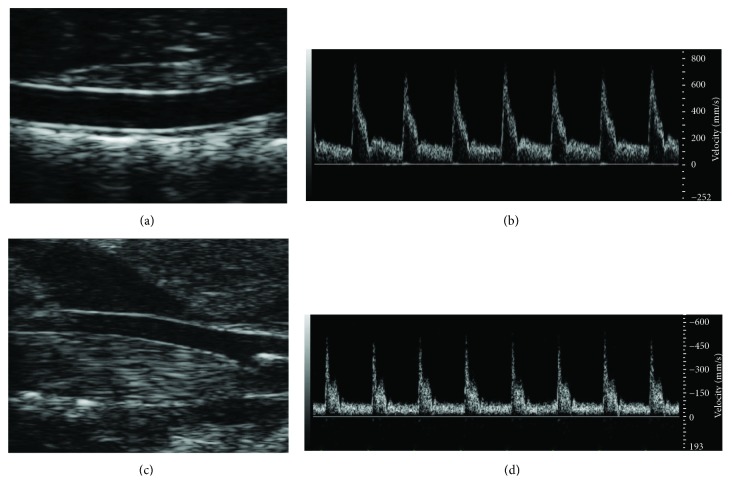
Abdominal aorta ultrasound scan. (a) B-mode image sequence was used to derived diameter and distension. (b) PW-Doppler signal was used to plot diameter-flow velocity loop thus evaluating PWV_abd_. Aortic pulse pressure was derived from PWV_abd_ and distension measurements. Furthermore, blood flow velocity signal was used to derive wall shear rate measurements. Carotid artery ultrasound scan. (c) B-mode image sequence was used to derived diameter and distension. (d) PW-Doppler signal was used to plot diameter-flow velocity loop thus evaluating PWV_car_. Furthermore, blood flow velocity signal was used to derive wall shear rate measurements.

B-mode of abdominal aorta (Figure 2(a)) and left common carotid (Figure 2(c)) were obtained with the lower and higher frequency probe, respectively; longitudinal views of both arteries were collected using the EKV™ (FUJIFILM VisualSonics Inc., Toronto, Canada) retrospective imaging mode, which allows a frame rate of 700 fps. Pulsed wave Doppler images (Figures 2(b) and 2(c)) were acquired immediately after the B-mode ones using the same scan projection, with the angle correction fixed at 60°. The pulse repetition frequency (PRF) was maintained at 20–25 kHz for the carotid artery and at 15–20 kHz for the abdominal aorta. B-mode and PW-Doppler images were postprocessed as described in [18]. Briefly, diameter instantaneous values were derived from B-mode images using edge detection and contour tracking techniques [19] while velocity data were derived obtaining the envelope of the PW-Doppler flow trace through an automatic method for the envelope detection. Mean diameter (Dm) and relative distension (relD) values were evaluated from the obtained diameter waveforms; relD was calculated as (D_s_−D_d_)/D_d_ and expressed as a percentage (D_s_ is the diameter in systole and D_d_ is the diameter in diastole). Pulse wave velocity (PWV), considered a surrogate marker for arterial stiffness [20], was assessed as previously described [18], starting from diameter and mean velocity values and using the diameter-velocity loop approach.

Mean diameter, relative distension, and pulse wave velocity values were calculated for both abdominal aorta and left carotid artery (Dm_abd_ and Dm_car_, relD_abd_ and relD_car_, and PWV_abd_ and PWV_car_).

Aortic pulse pressure (PP_ao_) was measured according the following equation: PP_ao_ = PWV_abd_^2^ · *ρ* · relD_abd_, where *ρ* = 1059 kg/m^3^ is the blood density.

The PP_ao_ equation was obtained inverting the well-known Bramwell-Hill relationship [21] between PWV, pulse pressure, and distension. A raw estimate of the wall shear rate for both carotid (WSR_car_) and abdominal (WSR_abd_) arteries was obtained according to 4 · V_mean_/D_d_ equation [22].

To test the possible effect of animal aging per se on our measurements, another ultrasound scan of the heart, abdominal aorta, and carotid, along with blood sampling, was repeated two months after basal evaluation in a small group of db/db mice (*N* = 6).

### 2.5. Renal Microcirculation Imaging and Assessment

**Figure 3:**
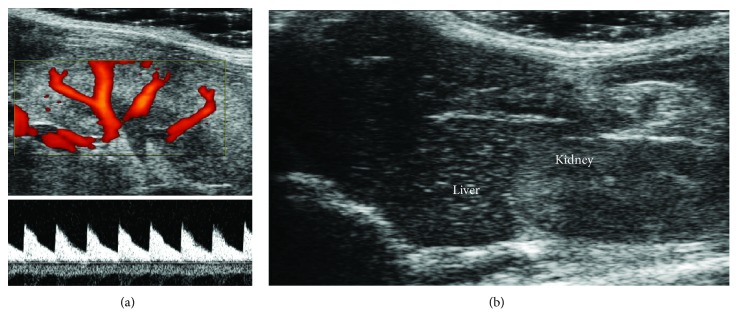
Kidney and liver ultrasound scan. (a) Power Doppler image was used to localize renal vessels, while PW-Doppler signal was used to measure renal blood flow thus deriving pulsatility and reflectivity indexes. (b) Mean gray levels of the liver parenchyma were compared to mean gray levels of the renal parenchyma.

A microultrasound examination of the left kidney was performed, and the renal vascular tree was mapped using power Doppler images (Figure 3(a)) acquired with a central frequency of 32 MHz and a PRF of 5 kHz. Renal flow velocity data were obtained placing the sample volume in correspondence of the central segmental artery and acquiring related PW-Doppler images (Figure 3(a)) with a PRF of 9-10 kHz. Peak systolic velocity (PSV) and end-diastolic velocity (EDV) values were manually measured; mean velocity (MV) was obtained from tracing the envelope of the flow signal correspondent to a single cardiac cycle. Renal resistivity index (RI) was calculated as (PSV-EDV)/PSV, while renal pulsatility index (PI) was assessed as (PSV-EDV)/MV.

Cardiac and renal evaluations were obtained with the software provided with the ultrasound equipment (VEVOLab, FUJIFILM VisualSonics Inc., Toronto, Canada). Vascular assessments required a customized images processing approach which was performed using Matlab R2011a (MathWorks Inc., Natick, MA, USA).

### 2.6. Liver Steatosis Assessment

An ultrasound B-mode scan of the liver was obtained with Vevo 2100 using the MS-250 24 MHz hfUS probe. Ultrasound projection was chosen in order to have also the right kidney in the same image (Figure 3(b)). Two regions of interest (ROIs) were placed in the liver and in the kidney parenchyma, respectively. The mean values of the gray levels of the two ROIs were evaluated and its ratio (that we call *steatoscore*) was considered as a surrogate index of steatosis degree.

### 2.7. Statistical Analysis

Data were analyzed with SPSS version 23 (IBM, New York, NY, USA) and are presented as median and interquartile range [IQR]. For all the evaluated parameters, the Mann–Whitney test was used for determining differences between wt and *db/db* mice. Tests were considered statistically significant when *p* < 0.05.

## 3. Results

Table 1 shows the phenotype of the two groups of animals. As expected, *db/db* mice were characterized by a higher weight and a significantly worse metabolic profile with the exception of cholesterol levels that were similar between *db/db* and wt mice. The phenotypic characteristics of the animals (triglycerides and AST not surprisingly elevated in *db/db* mice) agree with the literature and confirm the reliability of the model [23, 24].

Heart rate was significantly lower (296[77] versus 378[77] bpm, *p* < 0.01) in *db/db* mice. In addition, a small difference emerged in respiratory rate (134[53] in *db/db* versus 117[19] in wt, *p* = 0.031).

Ultrasonographic cardiac parameters are reported in Table 2. *db/db* mice showed several differences with respect to wt; in particular, they were characterized by a lower LV mass value (*p* < 0.01) and turned out to have reduced cardiac output (*p* < 0.0001), ejection fraction (*p* < 0.0001), fractional shortening (*p* = 0.001), and stroke volume (*p* < 0.0001). Concerning the diastolic function, a significant difference emerged from E/A evaluation (*p* < 0.0001), with *db/db* mice showing higher values than wt.

The strain analysis revealed a significant difference in radial strain (32.22[14.75]% in *db/db* versus 39.74[11.81]% in wt, *p* < 0.01) and strain rate of *db/db* and wt mice (7.88[3.31] s^−1^ in *db/db* versus 9.39[3.17] s^−1^ in wt, *p* = 0.02); all the others strain indexes were not different between the two groups.

As regards vascular evaluations, data are shown in Table 3. *db/db* mice were characterized by a lower mean aortic diameter (*p* = 0.001) and a lower relative distension of abdominal aorta and carotid arteries (*p* < 0.0001 and *p* < 0.01); carotid diameter and aortic and carotid pulse wave velocity did not differ between the two groups.

Both aortic and carotid wall shear rates were reduced in diabetic mice with *p* < 0.05 and *p* < 0.001, respectively.

Renal microcirculation analysis showed that both resistive index and pulsatility index were higher in *db/db* than in wt littermates (*p* < 0.01 for both, Table 4). Lastly, the liver steatosis index was higher in *db/db* mice but the difference did not reach the statistical significance with a borderline value of *p* = 0.059, Table 4.

There was no significant difference in all the ultrasound parameters acquired in a subgroup of 6 *db/db* mice two months after basal assessment (LVmass, CO, SV, FS, EF, E/A, Dm_abd_, relD_abd_, PWV_abd_, Dm_car_, relD_car_, and PWV_car_, all *p* > 0.05). Furthermore, no difference was noted in plasma cholesterol and glucose levels (all *p* > 0.05), while a significant increment was observed in triglycerides respect to basal values (*p* < 0.05).

## 4. Discussion

The main novelty of this paper is to offer for the first time a detailed characterization of the heart, liver, kidney, and vasculature phenotype of the *db/db* adult mouse as from its genetic defect, before the animal undergoes any dietetic or pharmacologic intervention aimed at affecting its metabolic profile for scientific purposes. The innovative ultrasonographic approach we have employed allows an *in vivo* multiorgan analysis through a safe and nonionizing imaging technique. Moreover, this technique induces a low level of stress in the animals and provides measurements of biomarkers under relatively physiologic conditions, requiring just a soft isoflurane-induced sedation for data acquisition. Finally, the high-frequency imaging technique has already demonstrated high accuracy and reliability, thus permitting the detection of even small differences across experimental groups.

Some interesting considerations can derive from our partially unexpected observations. A point of discussion is that, despite the higher weight, *db/db* animals show a reduced HR along with a low-performing myocardium both in diastolic and systolic phase: in fact, we did observe variations in E/A ratio, with absolute values greater than 2, thus suggesting the presence of a progressive diastolic dysfunction in these animals [25]. This effect can be attributed to the reduced physical activity [26] and an alteration in the cardiac autonomic balance of diabetic animals [27].

Furthermore, LV systolic function of *db/db* mice was impaired, with reduced SV, FS, EF, and CO values. Similar differences between diabetic and control animals were also confirmed by several authors [28–32] even though the absolute values of the biomarkers/parameters are quite variable in these reports, thus making difficult a direct comparison of the results.

The presence of a LV dysfunction was confirmed by strain analysis that shows a clear reduction of the radial strain and strain rate in *db/db* mice. A comparable result was partially confirmed by Li et al. [33]: using the same imaging technologies, they analyze the radial strain(rate) of *db/db* mice of three different ages (8–12–16 weeks), finding a significant difference only in the 16-week-old animals respect to age-matched controls. However, these authors limited the analysis of the cardiovascular health status uniquely to the strain imaging. Our animals entered into the study at the age of 13 weeks, and we could speculate that this time point was optimal in exacerbating the cumulative effects of persistent hyperglycemia, insulin resistance, and increased myocardial fat deposition on the onset of LV dysfunction to be detected through strain imaging.

Even more relevant appears the fact that these animals display a lower LV mass, thus demonstrating an absence of hypertrophic remodeling, despite the presence of myocardial dysfunction; this might resonate the restrictive cardiomyopathy described by Seferović et al. [34] in diabetic patients; however, those individuals were also characterized by a preserved ejection fraction.

Furthermore, van Bilsen et al. [25] associated the increased cardiac volumes only to the contemporary presence of sustained hypertension. Accordingly, we derived the aortic pulse pressure from the assessment of the local arterial stiffness, and we found an equivalent pulse pressure in the two groups, making questionable the role of the systemic pressure as major determinant of the after load and thus of LV mass in this animal model.

Broderick et al. [35] described a reduced heart weight in the 14-week-old *db/db* mice. We might speculate that a deleterious effect of sustained hyperglycemia and insulin resistance is able to impair the normal tissue and organ growth and development, inducing an early and progressive organ failure [36]. Alternatively, this failure might be related to a relative atrophy of the cardiac muscle as a result of an increased apoptosis [35, 37]. In any case, we are facing a model of dysfunctional heart different from the classic one, where a dilated and dysfunctional heart implies an augmented LV wall stress, which in turn can lead to a mass and volume increase, especially in the presence of aging and enhanced pre and after load [38]. This interpretation of our observations is supported by the fact that the two groups of animals show similar pulse pressure (PP_ao_) levels. Accordingly, other studies found no difference in pressure values (mean, systolic, diastolic, and pulse pressure) between *db/db* and wt mice [26, 39].

Our measurements of PP_ao_ seem to be lower than pulse pressure reported in the cited papers; this fact can be explained by the different physiological conditions between the telemetry-derived data acquired over 24 h (literature) and the PP_ao_ measured by us under anesthesia and at lower body temperature.

The observed difference in respiratory rate can be related to the reduced HR and cardiac output: a more frequent respiratory exchange of O_2_ can compensate the reduced amount of fresh circulating blood per beat.

Concerning the vascular assessment, we found a significant smaller aortic diameter and a similar aortic stiffness (evaluated as local aortic PWV) in *db/db* mice respect to wt; the same trend was confirmed at level of the carotid artery, but the difference in diameter did not reach the statistical significance. This phenotype, confirmed in diabetic patients at different arterial sites [40–42], can be ascribed to a reduced vessel tone secondary to a reduced NO bioavailability, in diabetic mice. In fact, these mice are also characterized by hyperleptinemia, frequently associated with endothelial dysfunction [43, 44]. Moreover, WSR_car_ and WSR_abd_ were significantly reduced in *db/db* mice compared to wt; this reduction means a lower mechanical stimulus to endothelial lining of the vessel, with a consequent reduced NO release.

No studies have, so far, explored renal vascular parameters in *db/db* mice *in vivo*. Renal resistive index, reflecting resistance and compliance of the renal vasculature, comes from a complex interaction between factors like renal interstitial pressure, peripheral vascular resistance, and systemic hemodynamics [45]. Our findings show a renal dysfunction, with both PI and RI indexes increased in *db/db* mice; this suggests for the first time the presence of a relatively early microvascular dysfunction in this animal model and makes *db/db* rodents suitable for studies aimed at improving the knowledge of renal damage during the course of the metabolic diseases. Chronic hyperglycemia and leptin resistance, by promoting the generation of systemic proinflammatory cytokines and shortage of NO, probably exert an array of direct and indirect influences on the renal vascular bed, involving both myogenic and tubule-glomerular feedback responses [46, 47]. This is relevant especially taking into account that the C57BL/6 strain is relatively resistant to the development of renal injury, including diabetic nephropathy [48].

Concerning liver function, our evaluation was just focused to detect and quantify the presence of steatosis in *db/db* mice. In this regard, the liver parenchyma is slightly brighter than the kidney parenchyma on ultrasound imaging (as demonstrated by the value of what we call the *steatoscore* parameter); this difference can be justified by a greater fat content in the liver respect to the kidney. However, the difference approaches only borderline the statistical significance (*p* = 0.059). In addition, this observation should be taken cautiously because the measurement of liver fat content by the *steatoscore* biomarker is still pioneering, being only the clinical ultrasound biomarker extensively validated against standard techniques. Of note, this study demonstrated that this technique is feasible and that a full validation against histological quantitative evaluation of fat liver content is desirable.

Finally, we tested the short-time changes of cardiovascular ultrasound biomarkers and lipids in a small group of *db/db* animals, finding difference only in the triglycerides levels. This fact is likely due to the sustained hyperglycemia, able to rapidly alter lipid profile without affecting the cardiovascular system in such a short time.

This study has some limitations. Measured values of the blood pressure are not reported. Tail cuff blood pressure analysis system, although available, was not used for two main reasons: first, the system operates fully in awake animals, mirroring a more stressed condition in comparison with that occurring during ultrasound imaging (sedated animals). Secondly, we experienced a very high variability in measurements of blood pressure obtained with this technique, discouraging the use of the device. Therefore, we prefer to assess a raw estimate of the pulse pressure inverting the Bramwell-Hill equation. Moreover, our pulse pressure assessment was done under anesthesia, as all imaging procedures, and high levels of pressure are not expected in the *db/db* model [26, 39] except when additional specific hits are included in the study design [25].

A second limitation of the study is the single time point analysis in the 13-week-old animals, which is, however, the typical entry point into the study for most experimental models. Finally, according to the high complexity of the disease in humans, the limited translatable insight of the murine *db/db* model should be recognized.

To conclude, the state-of-the-art ultrasound technology has been applied in the present work, choosing the image processing techniques to maximize reliability and repeatability of the results. By this approach, we provide for the first time a multiorgan, detailed characterization of the *db/db* mouse; our data can represent a useful tool for a more thoughtful choice of the right rodent model for any experimental design.

## Figures and Tables

**Table 1:** Weight and biochemical and physiological parameters in the two groups of mice.

	wt	*db/db*
Weight (g)	30 [2]	46 [5]^∗^
Blood glucose (mg/dl)	160 [31]	536 [73]^∗^
Cholesterol (mg/dl)	166 [47]	201 [81]
Triglycerides (mg/dl)	134 [38]	177 [45]^∗^
AST (IU/l)	53 [14]	96 [32]^∗^
HR^#^ (bpm)	378 [77]	296 [77]^∗^
RR^#^ (bpm)	117 [19]	134 [53] ^∗^

Data are presented as median [IQR]. ^∗^*p* < 0.05 versus wt. ^#^Data were obtained under anesthesia

**Table 2:** Ultrasonographic cardiac parameters in the two groups of mice.

	wt	*db/db*
LVmass (mg)	91 [13]	77 [17]^∗^
CO (ml/min)	13.49 [6.63]	7.01 [3.53]^∗^
SV (*μ*l)	38.88 [17.37]	22.82 [8.73]^∗^
FS (%)	14.80 [6.50]	9.24 [6.04]^∗^
EF (%)	53.50 [9.35]	44.24 [10.56]^∗^
E/A	1.37 [0.37]	2.00 [0.75]^∗^
gLS (%)	−14.39 [5.76]	−14.49 [4.36]
gLSR (1/s)	−4.54 [1.51]	−4.75 [3.35]
gRS (%)	39.74 [11.81]	32.22 [14.75]^∗^
gRSR (1/s)	9.39 [3.17]	7.88 [3.31]^∗^
gCS (%)	−23.63 [5.99]	−24.96 [5.61]
gCSR (1/s)	−8.18 [2.09]	−8.75 [2.75]

Data are presented as median [IQR]. ^∗^*p* < 0.05 versus wt. LVmass: left ventricular mass; CO: cardiac output; SV: stroke volume; FS: fractional shortening; EF: ejection fraction; E/A: E/A ratio; gLS/gLSR: global longitudinal strain/strain rate; gRS/gRSR: global radial strain/strain rate; gCS/gCSR: global circumferential strain/strain rate.

**Table 3:** Ultrasonographic vascular parameters in the two groups of mice.

	wt	*db/db*
Dm_abd_ (mm)	1.09 [0.12]	0.98 [0.11]^∗^
relD_abd_ (%)	20.77 [2.88]	17.39 [3.74]^∗^
PWV_abd_ (m/s)	1.80 [0.33]	1.90 [0.90]
Dm_car_ (mm)	0.45 [0.08]	0.42 [0.08]
relD_car_ (%)	23.29 [5.37]	19.20 [7.41]^∗^
PWV_car_ (m/s)	1.43 [0.57]	1.44 [0.53]
PP_ao_ (mmHg)	10.56 [4.57]	10.04 [9.08]
WSR_abd_ (1/s)	1039.00 [450.36]	929.09 [276.38]^∗^
WSR_car_ (1/s)	1630.56 [730.30]	991.82 [531.02]^∗^

Data are presented as median [IQR]. ^∗^*p* < 0.05 versus wt. Dm_abd_: abdominal aorta mean diameter; relD_abd_: abdominal aorta relative distension; PWV_abd_: abdominal aorta pulse wave velocity; Dm_car_: carotid artery mean diameter; relD_car_: carotid artery relative distension; PWV_car_: carotid artery pulse wave velocity; PP_ao_: aortic pulse pressure; WSR_abd_: abdominal wall shear rate; WSR_car_: carotid wall shear rate.

**Table 4:** Ultrasonographic hepatic and renal parameters in the two groups of mice.

	wt	*db/db*
RI	0.64 [0.12]	0.73 [0.12]^∗^
PI	1.02 [0.23]	1.23 [0.31]^∗^
*steatoscore*	0.79 [0.34]	0.95 [0.29]

Data are presented as median [IQR]. ^∗^*p* < 0.05 versus wt. RI: renal resistivity index; PI: renal pulsatility index; *steatoscore*: surrogate index of steatosis degree.
